# Optical Quantification of Cellular Mass, Volume, and Density of Circulating Tumor Cells Identified in an Ovarian Cancer Patient

**DOI:** 10.3389/fonc.2012.00072

**Published:** 2012-07-18

**Authors:** Kevin G. Phillips, Carmen Ruiz Velasco, Julia Li, Anand Kolatkar, Madelyn Luttgen, Kelly Bethel, Bridgette Duggan, Peter Kuhn, Owen J. T. McCarty

**Affiliations:** ^1^Department of Biomedical Engineering, School of Medicine, Oregon Health & Science UniversityPortland, OR, USA; ^2^Department of Cell Biology, The Scripps Research InstituteLa Jolla, CA, USA; ^3^Scripps Clinic Medical Group, Scripps ClinicLa Jolla, CA, USA; ^4^Department of Cell and Developmental Biology, School of Medicine, Oregon Health & Science UniversityPortland, OR, USA

**Keywords:** circulating tumor cell, ovarian cancer, differential interference contrast, quantitative phase microscopy, cellular mass, cellular volume, cellular density

## Abstract

Clinical studies have demonstrated that circulating tumor cells (CTCs) are present in the blood of cancer patients with known metastatic disease across the major types of epithelial malignancies. Recent studies have shown that the concentration of CTCs in the blood is prognostic of overall survival in breast, prostate, colorectal, and non-small cell lung cancer. This study characterizes CTCs identified using the high-definition (HD)-CTC assay in an ovarian cancer patient with stage IIIC disease. We characterized the physical properties of 31 HD-CTCs and 50 normal leukocytes from a single blood draw taken just prior to the initial debulking surgery. We utilized a non-interferometric quantitative phase microscopy technique using brightfield imagery to measure cellular dry mass. Next we used a quantitative differential interference contrast microscopy technique to measure cellular volume. These techniques were combined to determine cellular dry mass density. We found that HD-CTCs were more massive than leukocytes: 33.6 ± 3.2 pg (HD-CTC) compared to 18.7 ± 0.6 pg (leukocytes), *p* < 0.001; had greater volumes: 518.3 ± 24.5 fL (HD-CTC) compared to 230.9 ± 78.5 fL (leukocyte), *p* < 0.001; and possessed a decreased dry mass density with respect to leukocytes: 0.065 ± 0.006 pg/fL (HD-CTC) compared to 0.085 ± 0.004 pg/fL (leukocyte), *p* < 0.006. Quantification of HD-CTC dry mass content and volume provide key insights into the fluid dynamics of cancer, and may provide the rationale for strategies to isolate, monitor or target CTCs based on their physical properties. The parameters reported here can also be incorporated into blood cell flow models to better understand metastasis.

## Introduction

Ovarian cancer is the most lethal gynecological cancer and the fifth leading cause of cancer death among women in the United States. Over 90% of stage I patients with ovarian cancer can be cured with the current standard of care: tumor resection followed by platinum and taxane based chemotherapy. However, only 20% of ovarian cancers are detected in stage I (Bast et al., 2009). This is due in part to the anatomic location of the ovaries deep within the pelvis, the absence of tumor-specific symptoms (Lim et al., 2012) and a lack of effective serum markers that correlate with early disease progression (Anderson et al., 2010; Urban et al., 2011). The development of sensitive and specific diagnostic techniques for ovarian cancer is thus imperative to enhance the treatment outcomes of this disease. The enumeration and physical characterization of circulating tumor cells (CTCs) represents a new strategy to monitor the dynamics of tumor burden and potentially gain insight into the hematogenous transport of CTCs to other locations in the body.

Clinical studies have demonstrated that CTCs are present in the blood of cancer patients with known metastatic disease across the major types of epithelial malignancies (Allard et al., 2004). The concentration of CTCs in the blood is prognostic of overall survival in ovarian (Poveda et al., 2011), breast (Cristofanilli et al., 2004), prostate (Scher et al., 2009), colorectal (Cohen et al., 2008), and non-small cell lung cancer (Nieva et al., 2012) and is predictive of radiological response in some instances (Hayes et al., 2006; Cristofanilli et al., 2004). Further, case reports suggest that CTCs possess morphological features resembling the corresponding primary and/or metastatic lesions in breast (Marrinucci et al., 2007), colorectal (Marrinucci et al., 2009a), and lung (Marrinucci et al., 2009b) cancer. Together, these studies indicate that CTCs can be used to survey primary and metastatic lesions through minimally invasive peripheral blood draws or “fluid biopsies.”

While the morphology of CTCs is understood from the perspective of hematopathology, the physical properties of CTCs and their potential correlation to disease progression, the physics of hematogenous dissemination, and the processes governing arrest in distant organs are ill-defined. The observation that epithelial cells are less dense than red blood cells (RBCs) and tend to be large in comparison to hematopoetic cells has inspired the use of density dependent enrichment via centrifugation (Rosenberg et al., 2002) and size dependent selection through isolation by size filtration (Vona et al., 2000). In this study, we sought to measure the physical properties of CTCs without any fundamental assumptions regarding the value of these parameters. Hence, the detection technique is independent of these parameters. We utilized the high-definition (HD) CTC assay (Marrinucci et al., 2012) which retains all nucleated cells from peripheral blood and employs a rapid surface marker based fluorescence technique utilizing laser scanning microscopy to identify cytokeratin positive, CD45-negative, DAPI positive cells.

We analyzed 31 CTCs in a 66-year-old ovarian cancer patient with stage IIIC disease. The cells were isolated from a blood draw taken just prior to a debulking surgery. The HD-CTC assay is compatible with high magnification optical microscopy, enabling the use of non-interferometric quantitative phase microscopy (NI-QPM) techniques to measure cellular dry mass and density; as well as the use of differential interference contrast (DIC) microscopy to measure cellular volume. We present a comparison of these biophysical metrics among CTCs and normal leukocyte populations (*N* = 50) with the goal of understanding the cytophysical properties of cancer cells in the circulation. This study may provide the rationale for future strategies to isolate, target, and monitor CTCs using their physical properties.

## Materials and Methods

### HD-CTC enumeration and characterization

An ovarian cancer patient provided informed consent at Scripps Clinic (La Jolla, CA, USA) as approved by the Institutional Review Board. Eight milliliters of peripheral blood was collected in a rare cell blood collection tube (Streck, Omaha, NE, USA) and processed within 24 h after phlebotomy. HD-CTCs were identified according to the published protocol, the sensitivity and specificity of which is documented in Marrinucci et al. (2012). The technique consists of a red blood cell lysis, after which nucleated cells are attached as a monolayer to custom-made glass slides. These slides are the same size as standard microscope slides and possess a proprietary coating to ensure maximal retention of live cells. Slides were subsequently incubated with antibodies against cytokeratins (CK) 1, 4–8, 10, 13, 18, and 19 (Sigma); and CD45 with *Alexa* 647-conjugated secondary antibody (Serotec), nuclei were counterstained with DAPI (Serotec). For HD-CTC identification, an automated digital microscopy technique was used for fluorescence imaging. Potential CTCs were located and identified by computational analysis of the resulting image data. Fluorescence images of CTC candidates were then presented to a hematopathologist-trained technical analyst for interpretation. Cells were classified as HD-CTCs if they were CK-positive, CD45-negative, contained an intact DAPI nucleus without identifiable apoptotic changes or a disrupted appearance, and were morphologically distinct from surrounding leukocytes. Cartesian coordinates for each HD-CTC on a slide were generated from a fixed origin on that slide and used to relocate the cells of interest for NI-QPM and DIC measurements. Leukocytes located in the same field of view of HD-CTCs were chosen at random to be quantitatively compared to the HD-CTC population.

### Non-interferometric quantitative phase microscopy to determine cellular dry mass content

Due to their weak scattering and absorption properties over the optical spectrum, cells appear semi-transparent when imaged with standard brightfield microscopes. This low endogenous contrast under brightfield is a result of the amplitude of the waves traveling through the cell remaining relatively unchanged. The thickness and spatially variable density of the cell however, contributes to appreciable phase lags of the transmitted waves. This fact has inspired the utilization of phase as a contrast mechanism in cellular imaging in modalities such as phase contrast microscopy and DIC microscopy (Preza et al., 2011).

Under the paraxial approximation, appropriate to weak index contrast specimens such as cells, the phase, denoted Φ (radian), is defined as the sum of the relative refractive index of the sample along the (typically unknown) height of the specimen, denoted *h*(*x*, *y*) (μm) (Barer, 1952; Popescu, 2008)

$$\begin{array}{rc} \phi \left(x\text{,}\;y\right)\text{ = }\frac{\text{2}\pi }{\lambda }\int_{\text{0}}^{h\left(x,y\right)}\left[n_{\text{cell}}\left(x,y,z\right)-n_{0}\right]\text{dz} & \text{(1)} \end{array}$$

λ is the wavelength of light used when determining phase. Interestingly, Barer (1952) demonstrated that the dry mass content of a cell could be extracted from quantitative measurements of phase as a result of the cellular refractive index being linearly proportional to the dry mass content of a cell. This technique has been applied to the measurement cellular mass changes through the cell cycle (Mir et al., 2011).

Denoting by *C* (g/mL) the dry mass density of a cell, and α the specific refraction increment of the cell solids (∼0.2 mL/g), the refractive index is dependent on the dry mass density as given by

(2) $$n_{\text{cell}}(x,y,z)=n_{0}+\text{α}C(x,y,z).$$

Defining the “projected” mass density as $\text{ρ(}x,y)=\int_{0}^{h(x,y)}C(x,y,z)\text{ dz}\;(g/{\text{μm}}^{\text{2}}),$, we can obtain ρ from phase by substituting Eq. 2 into Eq. 1 to find

(3) $$\rho =\frac{\lambda \phi }{2\pi \alpha }\;\left[g∕\mu {\text{m}}^{2}\right]$$

Cell mass is then determined by integrating ρ over the area of the cell. Equation 3 requires no assumptions regarding the uniformity of the refractive index or mass density along height of the cell.

In the present investigation we utilize a computational technique based on the transport of intensity equation (TIE; Paganin and Nugent, 1998) that relates axial intensity, denoted *I*(*x, y, z*), variations to transverse phase variations

(4) $$-\frac{2\pi }{\lambda }\frac{\partial I\left(x,y,z\right)}{\partial z}=\nabla_{x,y}.\left[I\left(x,y,z\right)\nabla_{x,y}\phi \left(x,y,z\right)\right].$$

▽*_x,y_* denotes the two-dimensional gradient operator in the transverse *x*, *y* (perpendicular to optical, *z*-axis) coordinates. The TIE enables the determination of phase from standard brightfield intensity measurements acquired on a traditional microscope employing a charge coupled device (CCD) camera.

The TIE phase measurement consists of an image acquisition step and a post-processing procedure to determine phase. Low numerical aperture (NA) ∼0.3 Köhler illumination of the sample is carried out on a Zeiss Axio Imager 2 microscope (Carl Zeiss MicroImaging GmbH, Germany) with a green, λ = 540 ± 20 nm, filter (Chroma Technology Corp., Bellows Falls, VT, USA). Three-hundred through-focus brightfield intensity images of the sample are obtained with a 0.1-μm axial step size under software control by SlideBook 5.5 (Intelligent Imaging Innovations, Denver, CO, USA). The measured intensity is used to approximate the axial derivative of the intensity, left hand side of Eq. 4. A Green function technique is then utilized to solve for phase numerically (Frank et al., 2010) using a two-dimensional Fourier spectral method. Lastly, Eq. 3 is used to determine the projected mass density, ρ, from which the total cellular mass is determined by integration over the area of the cell.

### Preparation of polystyrene spheres for NI-QPM validation

To explore the validity of the NI-QPM technique we prepared polystyrene spheres whose phase properties are readily calculable. Twenty microlitres of a 10% solution of polystyrene spheres (Polysciences, Inc.) of diameter 0.11, 0.95, and 4.8 μm were pipetted separately on glass microscope slides (Fischer Scientific). Slides were air dried over night and then covered in Fluoromount G (Southern Biotech) whereupon a number 1.5 glass coverslip (Carl Zeiss MicroImaging) was placed overtop of the spheres. The sphere samples were stored overnight at 4°C to allow the Fluoromount G to cure. Prior to imaging, the samples were allowed to return to room temperature for 15 min.

### Differential interference contrast based cellular volume determination

HD-CTCs were relocated and through-focus DIC imagery at ×63 magnification, NA = 1.4, of each cell type was performed using a Zeiss Axio Imager 2 microscope (Carl Zeiss MicroImaging GmbH, Germany) with a green, λ = 540 ± 20 nm, filter (Chroma Technology Corp., Bellows Falls, VT, USA). Three-hundred planes through the sample were acquired with an axial increment of 0.1 μm. The microscope was under software control by SlideBook (Intelligent Imaging Innovations, Denver, CO, USA). Images were post-processed using a custom written program in MATLAB (The MathWorks, Inc., MA, USA). Post-processing consisted of the application of a Hilbert transform to each *en face* DIC image to ensure optimal contrast in image cube construction (Arinson et al., 2000). This process enables thresholding of DIC images at the cost of introducing some image blur along the axial direction. This low frequency noise was eliminated with the application of a high-pass filter to each sagittal plane of the image volume. The relative refractive index of the mounting media and immersion media (matching the cover glass) is low enough to prevent appreciable elongation of images along the optical axis (Rajadhyaksha et al., 1999). The cross sectional area of the cell in distinct sagittal planes was determined using a Sobel-based edge detection algorithm. Outlined sagittal planes of the cell separated by 0.6 μm were added together to obtain cellular volume. Each voxel in the analysis corresponds to a diffraction limited volume of 0.28 μm × 0.28 μm × 0.35 μm = 0.029 fL. Cellular area was determined by outlining each cell in *en face* DIC images.

### Statistical analysis

The Jarque–Bera test was used to evaluate normality of all parameters. One-way analysis of variance with Bonferonni *post hoc* analysis was used to assess statistical significance among parameters across multiple normally distributed cell parameters. The Kruskal–Wallis test was used to assess significance among non-normally distributed parameters. *P*-values of 0.05 or less were considered significant. All quantities presented as mean ± standard error of the mean unless otherwise noted.

## Results

### Transport of intensity based quantitative phase retrieval method maintains the resolution of the diffraction limit and is stable over two orders of magnitude of phase

To establish the accuracy of the TIE based phase retrieval algorithm employed in this study, we performed phase retrieval on polystyrene spheres (Polysciences, Inc., Warrington, PA, USA; *n*_sphere_ = 1.597, λ = 0.54 μm) mounted in Fluoromount G (Southern Biotech, Birmingham, AL, USA; *n*_Fl.G_ = 1.4) on glass slides. The reconstructed phase profile was compared to the theoretical phase profile for transmitted waves through a sphere of radius *r*, given by

(5) $$\phi =\frac{4\pi \left(n_{\text{sphere}}-n_{\text{Fl}\text{.G}}\right)}{\lambda }\sqrt{r^{2}-{\left(x-x_{0}\right)}^{2}}.$$

**Figure 1:**
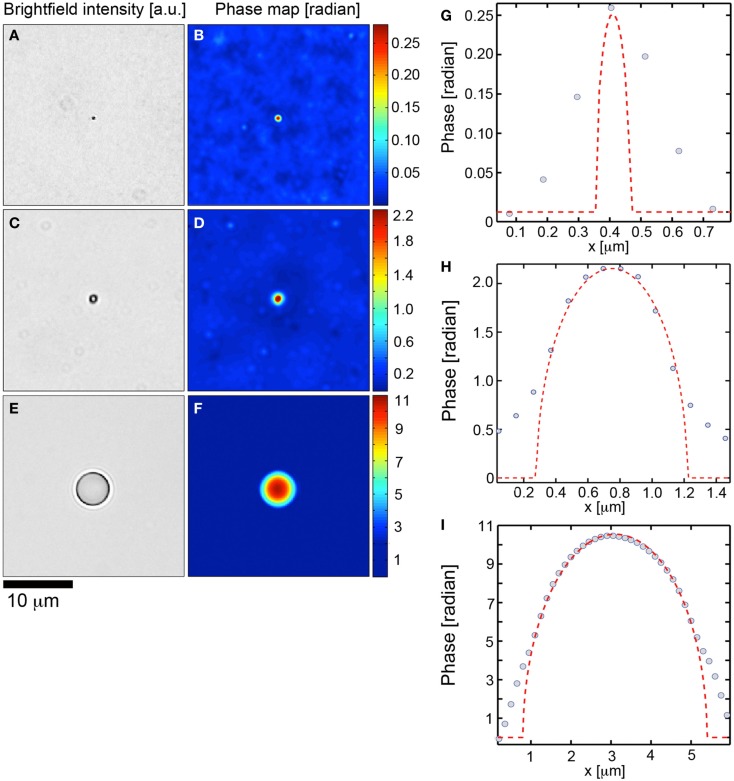
**Transport of intensity based quantitative phase microscopy validation over two orders of magnitude**. **(A,C,E)** Brightfield imagery of polystyrene spheres (*n* = 1.597, λ = 540 nm) suspended in fluoromount G (*n* = 1.4) with diameters of 0.11, 0.95, and 4.8 tm, respectively. **(B,D,F)** Corresponding transport of intensity based quantitative phase maps. **(G,H,I)** demonstrate theoretical phase profiles (dashed) for each polystyrene sphere with corresponding data (circles) overlaid.

First we investigated the ability of the TIE to perform phase retrieval at the diffraction limit of the microscope by evaluating the algorithm on 0.11 μm diameter spheres subject to Köhler illumination at an NA = 0.33. The measured resolution (×63, NA = 1.4) was 0.38 μm which agreed with the theoretical resolution for a system: 1.22λ/(1.4 + 0.33). A higher illumination NA could be used but at the expense of the validity of the TIE which is posed under the paraxial approximation. In Figures 1A,B we demonstrate the brightfield intensity and corresponding phase map. Figure 1G compares the phase profile through the center of the sphere to the theoretical profile. The TIE based phase had a full-width at half maximum spatial resolution of 0.39 μm. The maximal phase shift of the sphere is 0.25 ± 0.03 radians, given the manufacturer’s diameter variation of 0.012 μm, and was measured to be 0.26 by the TIE algorithm, within the manufacturing limits of the sphere. The diffraction limit of the microscope prevented any further information regarding the spatial dependence of the phase profile (Figure 1G).

We next investigated the phase retrieval over two orders of magnitude of the phase. Spheres of diameter 0.95 (Figures 1C,D,H) and 4.75 μm (Figures 1E,F,I) were investigated. The maximum theoretical phase shifts induced by the spheres were 2.18 ± 0.18, 10.9 ± 1.06 rad. In all cases, the TIE phase retrieval was able to recapitulate the theoretical values to within the manufacturing constrains of the spheres (Figures 1D,F). As well, the TIE method recapitulated the correct spatial phase profile inside the spheres (Figures 1H,I). Deviations of the TIE phase measurement at the edges of the spheres are to be expected from the discontinuity of the refractive index at these locations.

### HD-CTCs identified in an ovarian cancer patient have decreased densities, decreased mass, increased volume and increased area in comparison to normal peripheral leukocytes

**Figure 2:**
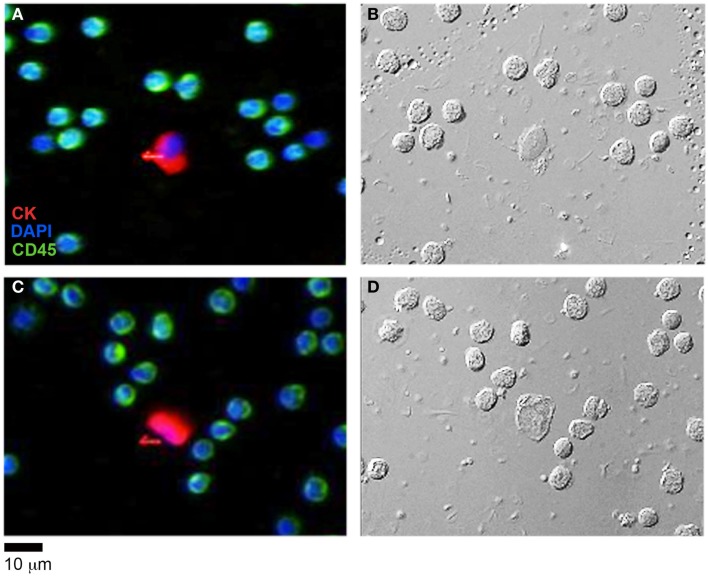
**HD-CTC identification of ovarian cancer CTCs and corresponding differential interference contrast (DIC) imagery**. **(A,C)** Immunofluorescent identification of HD-CTCs: CK+CD45−DAPI+ and peripheral leukocytes: CK−CD45+DAPI+ **(B,D)** corresponding differential interference contrast images of the same fields.

**Figure 3:**
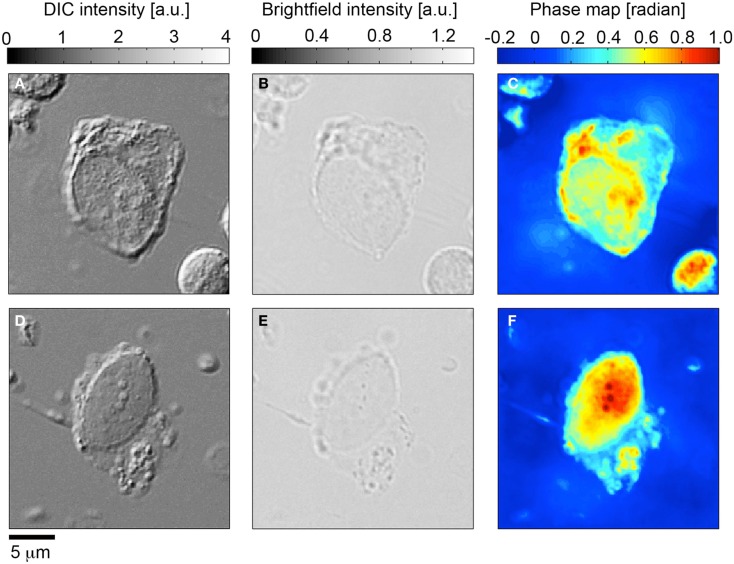
**Quantitative phase microscopy of circulating tumor cells; comparison to differential interference contrast (DIC) and brightfield microscopy**. **(A,D)** Differential interference contrast imagery of CK+CD45-DAPI+ HD-CTC5. **(B,E)** Corresponding bright field images. **(C,F)** Quantitative phase microscopy images determined from the transport of intensity equation analysis applied to brightfield through-focus imagery.

Having determined the validity of the TIE based NI-QPM algorithm, we performed phase imaging on 31 HD-CTCs. The HD-CTCs were identified in a single blood draw taken just prior to a debulking surgery from a 66-year-old ovarian cancer patient with stage IIIC disease. HD-CTCs were first identified by immunofluorescence staining and subsequently relocated for label-free analysis (Figure 2). Separate 25 μm × 25 μm × 30 μm image cubes of individual cells comprised of 18,750,000 voxels were created under brightfield and DIC settings (Figures 3A,B,D,E).

Brightfield image cubes were inputted into the TIE phase algorithm to determine phase profiles for each cell (Figures 3C,F). DIC image cubes were post-processed using a Hilbert transform technique to enable Sobel-based edge detection to quantify cellular volume.

**Figure 4:**
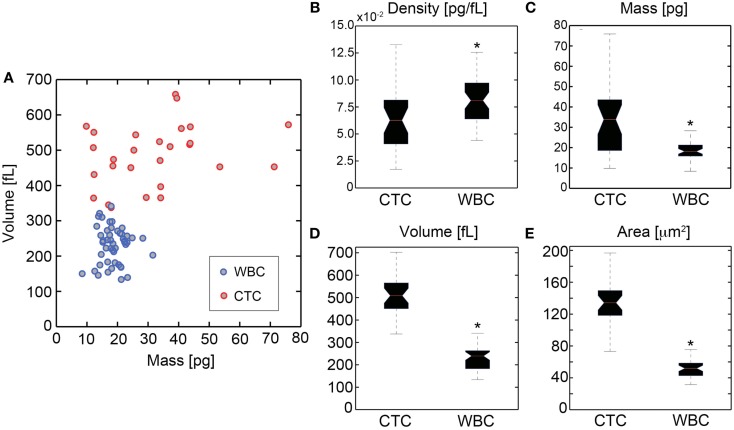
**Quantification of biophysical properties of ovarian cancer HD-CTCs**. **(A)** Scatter plot of cellular dry mass in pg (abscissa) versus cellular volume in fL (ordinate). Quantitative phase microscopy based mass measurements and differential interference contrast based volume measurements were uncorrelated. **(B)** Density box and whisker plot, **(C)** mass box and whisker, **(D)** volume box and whisker, **(E)** area box whisker. *Denotes *p* < 0.05 with respect to CTCs.

HD-CTCs and leukocytes separated into distinct populations in the mass-volume parameter space (Figure 4A) under the current sampling condition of similar numbers of leukocytes and HD-CTCs analyzed. We found that HD-CTCs were more massive than leukocytes (Table 1): 33.6 ± 3.2 pg (HD-CTC) compared to 18.7 ± 0.6 pg (leukocytes), *p* < 0.001 (Figure 4C) and had greater volumes (Table 1): 518.3 ± 24.5 fL (HD-CTC) compared to 230.9 ± 78.5 fL (leukocyte), *p* < 0.001 (Figure 4D). HD-CTCs were found to possess a decreased dry mass density with respect to leukocytes (Table 1): 0.065 ± 0.006 pg/fL (HD-CTC) compared to 0.085 ± 0.004 pg/fL (leukocyte), *p* < 0.006 (Figure 4B). CTC areas were larger than those of leukocytes (Table 1): 138.6 ± 8.1 μm^2^ (CTC) compared to 51.8 ± 1.5 μm^2^, *p* < 0.001 (Figure 4E).

**Table 1:** **Biophysical properties of normal peripheral leukocytes and ovarian cancer associated HD-CTCs**.

Cell type	Area (mm^2^)	Volume (fL)	Mass (pg)	Density (pg/fL)	*N*
Leukocyte (WBC)	51.8 ± 1.5	230.9 ± 78.5	18.7 ± 0.6	0.085 ± 0.004	50
HD-CTC	138.6 ± 8.1*	518.3 ± 24.5*	33.6 ± 3.2*	0.065 ± 0.006*	31

Area was determined from outlining the boarder of cells using DIC imaging. Volume was determined using a Hilbert transform post-processing algorithm on through-focus DIC image cubes. A non-interferometric quantitative phase microscopy method was used to determine cellular dry mass content. Cellular density was determined by dividing cellular mass by cellular volume. *Denotes *p* < 0.05 with respect to leukocytes.

## Discussion

Quantification of the biophysical properties of CTCs provides insight into the physics of the fluid phase of cancer. Flow-dependent interactions with RBCs in the vessels of the microcirculation give rise to the prevalence of less dense cells along the periphery of the vessel wall (e.g., platelets, leukocytes), a phenomenon known as margination (Goldsmith and Spain, 1984). Oxygenated RBCs have a dry mass density of 0.3 pg/fL (Park et al., 2008) by contrast, the leukocytes and HD-CTCs analyzed in this study are roughly 3.5–4.5 times less dense than RBCs, respectively (Table 1). The reduced dry mass density of leukocytes and HD-CTCs in comparison to RBCs suggests that these less dense cells may be physically trafficked to the vessel periphery through margination in a similar manner. Proximity of CTCs to the endothelium coupled with a large surface area could enhance the kinetics for interactions with endothelial cells and aid in promoting metastasis.

The label-free optical measurement of dry mass density and volume of HD-CTCs and leukocytes quantifies the density overlap and volumetric differences among these cells. Area and volume were the most statistically significant features that separated the populations from a physical standpoint. The dry mass density values reported here (Table 1) demonstrate why CTC enrichment using a density-based method is prone to leukocyte contamination (Rosenberg et al., 2002): the overlap of leukocytes and HD-CTCs makes the use of this parameter an unlikely means to separate the two cell populations (Figure 4B). Volumetric differences among these cell types demonstrate that isolation by size methods would omit a fraction of leukocytes (assuming an 8 μm diameter filter) but again, at the cost of leukocyte contamination. One may be tempted to make the filter size larger, allowing more leukocytes to pass through the filter. This should result in reduced leukocyte contamination but at the cost of omitting the potential CTC subpopulation that could overlap volumetrically with leukocytes. This preliminary study in an Ovarian cancer patient demonstrated that volume could separate the CTCs from the leukocyte population. However, more investigations across patients, tumor types, and treatment regimens are required to ensure members of the CTC population would not be lost in the use of larger filter sizes in filtration enrichment approaches. The HD-CTC assay, an enrichment free method, is an optimal tool to investigate the rationale for biophysical based enrichment strategies for CTC detection and lab-on-a-chip type characterizations.

The biophysical properties of HD-CTCs offer quantitative metrics with which to document potential changes in the CTC population in response to therapeutics, disease progression, or interventional surgeries. This label-free biophysical characterization can be carried out in parallel with current efforts to understand the genetic and proteomic composition of both the solid and fluid phase of caner. Together, these complementary approaches might aid in the search for targeted therapies.

## Conflict of Interest Statement

The HD-CTC technology has been licensed to Epic Sciences. Authors of this manuscript have ownership in Epic Sciences.
